# Risk of chronic kidney disease in 260 patients with lupus nephritis: analysis of a nationwide multicentre cohort with up to 35 years of follow-up

**DOI:** 10.1093/rheumatology/keae236

**Published:** 2024-04-22

**Authors:** Filipa Farinha, Sofia Barreira, Maura Couto, Margarida Cunha, Diogo Fonseca, Raquel Freitas, Luís Inês, Mariana Luís, Carla Macieira, Ana R Prata, Joana Rodrigues, Bernardo Santos, Rita Torres, Ruth J Pepper, Anisur Rahman, Maria J Santos

**Affiliations:** Centre for Rheumatology, University College of London, London, United Kingdom; Serviço de Reumatologia e Doenças Ósseas Metabólicas, Centro Hospitalar e Universitário de Lisboa Norte, Centro Académico de Medicina de Lisboa, Lisboa, Portugal; Rheumatology Department, Centro Hospitalar Tondela-Viseu, Viseu, Portugal; Rheumatology Department, Hospital Garcia de Orta, Almada, Portugal; Rheumatology Department, Centro Hospitalar de Vila Nova de Gaia/Espinho, Vila Nova de Gaia, Portugal; Rheumatology Department, Hospital Garcia de Orta, Almada, Portugal; Rheumatology Department, Centro Hospitalar e Universitário de Coimbra, Coimbra, Portugal; Rheumatology Department, Centro Hospitalar e Universitário de Coimbra, Coimbra, Portugal; Serviço de Reumatologia e Doenças Ósseas Metabólicas, Centro Hospitalar e Universitário de Lisboa Norte, Centro Académico de Medicina de Lisboa, Lisboa, Portugal; Rheumatology Department, Centro Hospitalar e Universitário de Coimbra, Coimbra, Portugal; Serviço de Reumatologia, Unidade Local de Saúde do Alto Minho, Ponte de Lima, Portugal; Rheumatology Department, Centro Hospitalar do Baixo Vouga, EPE, Aveiro, Aveiro, Portugal; Serviço de Reumatologia, Centro Hospitalar Lisboa Ocidental—Hospital Egas Moniz, Lisboa, Portugal; Centre for Nephrology, University College London, London, United Kingdom; Centre for Rheumatology, University College of London, London, United Kingdom; Rheumatology Department, Hospital Garcia de Orta, Almada, Portugal; Unidade de Investigação em Reumatologia, Instituto de Medicina Molecular, Faculdade de Medicina, Universidade de Lisboa, Centro Académico de Medicina de Lisboa, Lisboa, Portugal

**Keywords:** systemic lupus erythematosus, membranous lupus nephritis, proliferative lupus nephritis, chronic kidney disease, survival analysis

## Abstract

**Objectives:**

To compare proliferative (PLN) and membranous (MLN) lupus nephritis (LN) regarding clinical and laboratory presentation and long-term outcomes, and to investigate predictors of progression to chronic kidney disease (CKD).

**Methods:**

Multicentre observational study, with retrospective analysis of a prospective cohort, using data from the Rheumatic Diseases Portuguese Registry – Reuma.pt. Patients with biopsy-proven PLN, MLN and mixed LN were included. Cox regression survival analysis was used to investigate predictors of CKD.

**Results:**

A total of 260 patients were included. Median follow-up was 8 years (IQR 11; minimum 1, maximum 35 years). MLN patients presented with significantly lower serum creatinine [0.70 (IQR 0.20; minimum 0.50, maximum 1.30) mg/dl *vs* 0.80 (IQR 0.31; minimum 0.26, maximum 2.60) in PLN, *P* = 0.003]. Proteinuria levels did not differ between groups (*P* = 0.641). Levels of complement were reduced in PLN but nearly normal in MLN patients, and there were fewer patients with positive anti-dsDNA antibodies in the MLN group (*P* < 0.001). One year after the beginning of treatment, 62% of the patients achieved EULAR/ERA-EDTA complete response, with a further 5% achieving partial response. Patients with lower proteinuria at diagnosis were more likely to achieve a complete renal response at one year; however, proteinuria at diagnosis or at one year did not predict long-term CKD. Estimated glomerular filtration rate (eGFR) ≤75 mL/min/1.73 m^2^ at one year was the strongest predictor of progression to CKD (HR 23 [95% CI 8–62], *P* < 0.001). Other possible predictors included the use of azathioprine for induction of remission, older age at diagnosis and male sex.

**Conclusion:**

Proteinuria levels did not predict LN histologic class in our cohort. eGFR cutoff of 75 mL/min/1.73 m^2^ after one year of treatment was strongly predictive of progression to CKD.

Rheumatology key messages

- Levels of proteinuria did not predict whether the kidney biopsy would show MLN or PLN.
- MLN has a different serological profile than PLN, possibly reflecting different pathogenesis.
- eGFR ≤75 ml/min/1.73 m^2^ at one year was the best predictor of progression to CKD.

## Introduction

SLE is a challenging multisystem autoimmune disease that frequently affects the kidneys. LN is one of its most serious manifestations and occurs in up to 50% of the patients [1–3]. Although there has been a significant improvement in survival of patients with SLE and LN over the past decades [4, 5], LN is still associated with progression to end-stage renal disease (ESRD) and death [1, 6].

LN is classified based on the renal biopsy according to the 2003 International Society of Nephrology/Renal Pathology Society (ISN/RPS) classification system [7], which was revised in 2018 [8]. Over 50% of patients have either a Class III or Class IV proliferative LN (PLN), with 20% to 30% demonstrating membranous LN (MLN) [9, 10]. Around 15% of patients show a mixed pattern of membranous and proliferative histologic changes [1, 7].

A number of studies have investigated the long-term outcomes of patients with PLN [11–13], MLN [14–16], and some studies have compared pure PLN or pure MLN *vs* mixed LN [17–20]. We have recently analysed a single-centre cohort of patients with LN from the UK, with up to 42 years of follow-up, and reported on long-term outcomes and differences between PLN and MLN [21]. However, to our knowledge, there are no other studies directly comparing PLN *vs* MLN. A multicentre study, with a different population, would be important to confirm and validate our previous findings.

Therefore, our objectives were to compare patients with MLN and PLN regarding clinical and laboratory presentation and long-term outcomes, and to investigate predictors of progression to chronic kidney disease (CKD) in a nationwide multicentre cohort of patients with LN.

## Methods

### Study population

This is a multicentre observational study, with retrospective analysis of a cohort, using data from the Rheumatic Disease Portuguese Registry – Reuma.pt. It is an observational, long-term national registry, conceived and promoted by the Portuguese Society for Rheumatology. It started in 2008 and began enrolling patients with SLE prospectively in 2012, with retrospective data included before 2012. After obtaining informed consent from the patient, data was abstracted as an electronic clinical file [22, 23].

Study subjects had to fulfil ACR 1997 [24] or SLICC 2012 [25] classification criteria for SLE, with a renal biopsy showing LN class III, IV, V or a combination of these, according to the ISN/RPS 2003 classification system [7]. Exclusion criteria included a minimum duration of follow-up less than one year. Patients included in our study were enrolled from 10 different clinical sites. We divided the patients into three groups, according to the classification of the first renal biopsy: PLN (class III and IV), MLN (class V) and mixed LN (III+V or IV+V).

Patients were not involved in the design of the study.

This study complies with the Declaration of Helsinki and was approved by the Scientific Board of Reuma.pt. We have ethical approval from the Institute of Child Health/Great Ormond Street Hospital Research Ethics Committee (05/Q0508/6). Written informed consent was obtained from all participants in the registry Reuma.pt.

### Data collection

#### Demographic data: year of birth, sex, ethnicity

Clinical data: Year of diagnosis of SLE, classification criteria fulfilled; date of diagnosis of nephritis, number of renal biopsies, date of each renal biopsy, class of LN in each biopsy, development of CKD, ESRD, renal transplant; Systemic Lupus Erythematosus Disease Activity Index-2000 (SLEDAI-2K) at the time of LN diagnosis and at 12 months after diagnosis. Year of last visit, year and cause of death (if occurred); treatment with antimalarials, immunosuppressants, corticosteroids, renin–angiotensin–aldosterone system blockers and non-steroidal anti-inflammatory drugs. Comorbidities: antiphospholipid syndrome, diabetes mellitus and arterial hypertension.

Laboratory data: autoantibody profile: ever-positive (according to the cut-off of each centre’s laboratory) ANA, anti-dsDNA, anti-Sm, anti-RNP, anti-Ro, anti-La; antiphospholipid antibodies (anti-cardiolipin, anti-beta2glycoprotein1, lupus anticoagulant); ever-low C3 or C4; urinary protein/creatinine ratio (uPCR) or 24 h-proteinuria, serum creatinine, albumin, positivity for anti-dsDNA, C3, C4, ESR and CRP, all at the time of biopsy and 12 months afterwards, and at the time of last visit. As different centres use different methods to quantify anti-dsDNA antibodies, the level of antibodies is not comparable between centres; therefore, we included anti-dsDNA only as a binary variable: positive or negative according to the cut-off of each centre’s laboratory.

We defined renal response according to the EULAR/ERA-EDTA (European League Against Rheumatism/European Renal Association–European Dialysis and Transplant Association) definition [26], as follows:

Complete response: proteinuria <500–700 mg/24 h with (near) normal eGFR. We accepted an eGFR above 81 mL/min/1.73 m^2^ (10% below normal) as near normal.

Partial response: decrease in proteinuria by at least 50%, to subnephrotic levels, with (near) normal eGFR, above 81 mL/min/1.73 m^2^.

#### No response: all other cases

We defined CKD as decreased GFR below 60 mL/min/1.73 m^2^ for at least 3 months, according to the Kidney Disease: Improving Global Outcomes (KDIGO) guidelines. We determined the estimated glomerular filtration rate (eGFR) for all patients using the CKD-EPI creatinine 2009 equation [27].

### Statistical analysis

Statistical analysis was performed with IBM^®^ SPSS^®^ statistics version 22. PLN and MLN groups were compared using Pearson’s χ^2^ or Fisher’s exact test for categorical variables and *t* test or Mann–Whitney for numerical variables, as appropriate. We reported all of the individual non-corrected *P* values and afterwards a Bonferroni correction was applied, to account for multiple comparisons.

Cox regression analysis was used to investigate predictors of CKD. Cases with missing data were omitted. We started by testing the following variables with univariable Cox regression: sex, ethnicity, age at diagnosis, clinical manifestations (the ones included in classification criteria), laboratory measures at diagnosis and at one-year, historical serology, comorbidities, treatment and response at one year. For continuous numerical variables showing a significant result, we constructed ROC curves to investigate whether there was a cut-off for each variable, which could be a good predictor of the event. We then tested that cut-off in the Cox regression, to find its hazard ratio. Afterwards, the variables showing significant results were tested together, using a maximum of one covariate for every 10 events in the multivariable Cox regression model. Kaplan–Meier curves were drawn. Significance level was set at 0.05.

## Results

### Patients with PLN have higher creatinine levels, more frequent positivity for anti-dsDNA antibodies and lower complement levels than those with MLN, at time of renal biopsy

A total of 260 patients with biopsy-proven PLN [*n* = 203 (71% with class IV and 29% with class III)]), MLN (*n* = 47) or mixed LN (*n* = 10) were included in this study. Median age at the time of renal biopsy was 30 years (IQR 18; minimum 11, maximum 73 years) and median time between diagnosis of SLE and LN was one year (IQR 5; minimum 0, maximum 26 years). The median total duration of follow-up since the diagnosis of LN was 8 years (IQR 11; minimum 1, maximum 35 years).

As seen in Table 1, among patients with MLN, there was a higher proportion of individuals with African ancestry (17.4%) and a lower proportion of White Europeans (78.3%) compared with the PLN group (8.5 and 90%, respectively); however, this difference did not reach statistical significance (*P* = 0.083).

**Table 1.** Characteristics of the Reuma.pt cohort of patients with proliferative, membranous and mixed LN, and comparison between proliferative and membranous LN

		Total N = 260	Class III and IV N = 203	Class V N = 47	*P*
Females, N (%)		225 (87)	174 (86)	45 (96)	0.060
Ethnicity					
White European, N (%)		224 (88)	180 (90)	36 (78.3)	0.083
African ancestry, N (%)		27 (10)	17 (8.5)	8 (17.4)	
Other, N (%)		5 (2)	3 (1.5)	2 (4.3)	
Time SLE-LN(y), median (IQR)		1 (5)	1 (5)	1 (6)	0.734
Initial-onset LN, N (%)		112 (43)	91 (45)	17 (38)	0.388
Age LN diagnosis(y), median (IQR)		30 (18)	30 (19)	31 (14)	0.597
SLEDAI-2K at LN diagnosis, median (IQR)		15 (9)	16 (10)	11 (10)	**0.008**
Laboratory at LN diagnosis		N’=169	N’=135	N’=26	
uPCR, median (IQR)		1700 (2465)	1650 (2437)	1644 (2067)	0.641
Nephrotic-range proteinuria, N (%)		40 (24)	32 (24)	5 (19)	0.620
Creatinine, median (IQR)		0.80 (0.33)	0.80 (0.31)	0.70 (0.20)	**0.003**
eGFR, mean ± SD		99 ± 32	98 ± 32	112 ± 20	**0.002**
eGFR <60, N (%)		22 (12)	16 (11)	1 (3)	0.312
Albumin, mean ± SD		33 ± 10	33 ± 7	34 ± 6	0.707
ESR, median (IQR)		29 (43)	28 (41)	34 (44)	0.302
CRP, median (IQR)		0.34 (1.16)	0.38 (1.29)	0.27 (0.51)	0.191
C3, mean ± SD		0.68 ± 0.28	0.65 ± 0.26	0.85 ± 0.33	<0.001*
C4, median (IQR)		0.10 (0.11)	0.09 (0.09)	0.14 (0.11)	**0.001**
Positive anti-dsDNA, N (%)		140 (85)	121 (92)	12 (48)	<0.001*
Other manifestations, N (%)					
Mucocutaneous		178 (69)	137 (68)	33 (70)	0.718
Joint involvement		179 (70)	139 (69)	30 (65)	0.604
Serositis		87 (34)	71 (36)	12 (27)	0.241
Neurologic		18 (8)	14 (7)	3 (7)	1.000
Haematological		189 (76)	154 (79)	26 (62)	**0.022**
Historical serology (ever positive), N (%)					
ANA		251 (97)	197 (98)	44 (96)	0.617
Low complement		219 (89)	175 (91)	34 (79)	**0.031**
Anti-dsDNA		235 (91)	190 (94)	36 (80)	**0.005**
Anti-Sm		59 (24)	42 (22)	14 (33)	0.153
Anti-Ro		84 (35)	62 (33)	19 (45)	0.145
Anti-La		34 (14)	26 (14)	6 (14)	0.959
Anti-RNP		74 (32)	52 (28)	20 (48)	**0.016**
Antiphospholipid antibodies		69 (35)	54 (35)	12 (35)	0.940
Antiphospholipid syndrome, N (%)		16 (7)	11 (6)	5 (13)	0.166
Hypertension (ever), N (%)		59 (26)	47 (26)	7 (18)	0.315
Diabetes (ever), N (%)		9 (4)	8 (4)	1 (6)	1.000

There were missing data regarding laboratory at LN diagnosis, therefore N’ represents the number of patients included in the analysis.

* *P* values <0.001 remain significant after applying the Bonferroni correction.

eGFR: estimated glomerular filtration rate, mL/min/1.73 m^2^; ESRD: end-stage renal disease; LN: lupus nephritis; SLE: systemic lupus erythematosus; uPCR: urinary protein-creatinine ratio, mg/g; y: years. Creatinine is presented in mg/dl, albumin in g/L and C3 in g/L. The *P*-value refers to the comparison between PLN and MLN: the mixed LN group, included in the total, was excluded from the comparison due to small sample size (only 10 patients).

*P* values < 0.05 are presented in bold.

The proportion of involvement of other organs and systems is also shown in Table 1. Patients with PLN presented a higher frequency of haematological manifestations (79% *vs* 62%, *P* = 0.022).

Only 9 out of 260 patients (4%) had diabetes and 59 (26%) had hypertension. Antiphospholipid syndrome was present or developed in 16 patients (7%), although positivity for antiphospholipid antibodies was more frequent: 69/260 (35%). Frequency of diabetes, hypertension, antiphospholipid syndrome and antiphospholipid antibodies did not differ between PLN and MLN groups.

Serum creatinine levels at the time of renal biopsy were significantly higher in patients with PLN, 0.80 mg/dl (IQR 0.31; minimum 0.26, maximum 2.60) *vs* 0.70 mg/dl (IQR 0.20; minimum 0.50, maximum 1.30) in those with MLN (*P* = 0.003). This translated into a significantly lower eGFR in patients with PLN (98 ± 32 *vs* 112 ± 20 mL/min/1.73 m^2^, *P* = 0.002). Within PLN, patients with class IV had lower eGFR than patients with class III, although the difference did not reach statistical significance (93 ± 33 *vs* 104 ± 30 mL/min/1.73 m^2^, *P* = 0.064).

Interestingly, the level of proteinuria did not differ between groups (*P* = 0.641), with a median uPCR of 1700 mg/g (IQR 2465; minimum 440, maximum 18537). A total of 24% and 19% of patients with PLN and MLN, respectively, had nephrotic range proteinuria (*P* = 0.620). Similarly, serum albumin levels did not differ significantly and were mildly reduced in both groups: 33 ± 7 g/l in PLN and 34 ± 6 g/l in MLN (*P* = 0.707).

There was no association between ethnicity/race and the levels of uPCR (*P* = 0.776) or serum creatinine (*P* = 0.269) at diagnosis.

At the time of the renal biopsy, levels of complement C3 and C4 were reduced in PLN (C3 = 0.65 ± 0.26 g/l) but nearly normal in MLN patients (C3 = 0.85 ± 0.33 g/l, *P* < 0.001), as seen in Table 1. Furthermore, only half of the patients with MLN showed positivity for anti-dsDNA antibodies (48%) compared with 92% in the PLN group (*P* < 0.001).

Differences in serology persisted between these two groups, during the course of the disease. In fact, when we consider ever-positivity for anti-dsDNA, this was still lower in the MLN group (80% *vs* 94% in the PLN group, *P* = 0.005). Ever-low C3 and/or C4 was also less frequent in MLN patients (79% *vs* 91%, *P* = 0.031). Conversely, the MLN group showed a higher proportion of patients with ever-positive anti-RNP antibodies (48% *vs* 28%, *P* = 0.016).

Disease activity at the time of biopsy, as assessed by the SLEDAI-2K for a 30-day window [28], was higher in patients with PLN (median 16, IQR 10) than in those with MLN (median 11, IQR 10, *P* = 0.008).

### Two-thirds of the patients showed complete or partial renal response at one year

Ninety-nine percent of patients with PLN and 81% of those with MLN received induction treatment with a combination of immunosuppressants and steroids. Details on agents used are shown in Table 2. Pulses of IV methylprednisolone were used in 20% of patients with PLN and 7% of patients with MLN.

**Table 2.** Treatment and outcomes of the Reuma.pt cohort of patients with proliferative, membranous and mixed LN, and comparison between proliferative and membranous LN

	Total N = 260	Class III and IV N = 203	Class V N = 47	*P*
Induction treatment, N (%)	N’=209	N’=158	N’=42	
Mycophenolate	102 (49)	80 (51)	18 (43)	0.370
Cyclophosphamide	66 (32)	62 (39)	0 0	<0.001*
Azathioprine	14 (7)	9 (6)	4 (9.5)	0.478
Rituximab	6 (3)	4 (2.5)	0 (0)	0.581
Calcineurin inhibitors	13 (6)	1 (0.6)	12 (29)	<0.001*
Maintenance treatment, N (%)				
Mycophenolate	121 (59)	101 (65)	16 (38)	**0.002**
Cyclophosphamide	15 (7)	15 (10)	0 (0)	**0.044**
Azathioprine	32 (16)	26 (17)	3 (7)	0.121
Rituximab	2 (1)	2 (1.3)	0 (0)	1.000
Calcineurin inhibitors	15 (7)	1 (0.6)	13 (31)	<0.001*
Use of antimalarials, N (%)	234 (94)	184 (94)	41 (93)	0.742
Use of steroids, N (%)	208 (86)	160 (85)	39 (89)	0.501
Use of ACEi/ARB, N (%)	186 (75)	145 (75)	36 (84)	0.210
Use of NSAIDs (ever), N (%)	23 (9)	18 (9)	5 (11)	0.777
EULAR/ERA-EDTA response at one year	N’’ = 147	N’’ = 117	N’’ = 26	
Complete renal response, N (%)	92 (62)	78 (67)	13 (50)	0.098
Partial renal response, N (%)	7 (5)	4 (4)	2 (8)	0.306
No response	48 (33)	33 (29)	11 (42)	0.176
SLEDAI-2K at one year, median (IQR)	4 (6)	4 (4)	2 (9)	0.620
Laboratory at one year				
uPCR, median (IQR)	192 (530)	172 (481)	200 (1181)	0.121
Creatinine, median (IQR)	0.76 (0.26)	0.76 (0.23)	0.69 (0.47)	0.310
eGFR, mean ± SD	104 ± 30	105 ± 28	107 ± 27	0.646
Time follow-up since LN, median (IQR)	8 (11)	10 (11)	7 (10)	0.272
Laboratory at the last visit				
uPCR, median (IQR)	110 (235)	111 (245)	111 (225)	0.773
Creatinine, median (IQR)	0.75 (0.26)	0.75 (0.32)	0.76 (0.15)	0.358
eGFR, median (IQR)	100 (38)	102 (41)	100 (30)	0.503
>1 renal biopsy, N (%)	42 (16)	37 (18)	5 (11)	0.210
Different class in subs. biopsy, N (%)	12 (29)	9 (24)	3 (60)	0.131
CKD after LN diagnosis, N (%)	38 (16)	32 (17)	3 (7)	0.104
ESRD after LN diagnosis – total, N (%)	10 (4)	7 (4)	1 (2)	1.000
ESRD – subgroup transplant, N (%)	6 (60)	3 (43)	1 (100)	1.000
Deaths, N (%)	16 (6)	15 (7)	1 (2)	0.319

There were missing data for some variables, therefore N’ represents the number of patients analysed regarding treatment and N’’ the number of patients analysed regarding EULAR/ERA-EDTA response at one year.

* *P* values <0.001 remain significant after applying the Bonferroni correction.

ACEi/ARB: angiotensin-converting enzyme inhibitors/angiotensin receptor blockers; CKD: chronic kidney disease (eGFR < 60 mL/min/1.73 m^2^); eGFR: estimated glomerular filtration rate, mL/min/1.73 m^2^; ESRD: end-stage renal disease; LN: lupus nephritis; NSAIDs: nonsteroidal anti-inflammatory drugs; SLEDAI: Systemic Lupus Erythematosus Disease Activity Index; uPCR: urinary protein-creatinine ratio, mg/g; y: years. Creatinine is presented in mg/dl, albumin in g/L and C3 in g/L.

*P* values < 0.05 are presented in bold.

One year following the renal biopsy and the beginning of treatment, median uPCR decreased from 1700 to 192 mg/g. Serum creatinine decreased in patients with PLN and by one year there was no significant difference between groups (0.76 mg/dl in PLN *vs* 0.69 mg/dl in MLN, *P* = 0.310).

According to the EULAR/ERA-EDTA definition, 62% of the patients achieved complete response at one year, with a further 5% achieving a partial response. One-third of the patients had not responded to treatment at one year. Patients with lower proteinuria at diagnosis were more likely to achieve a complete renal response at one year. The uPCR at diagnosis was 1357 (IQR 1782) mg/g in patients who achieved a complete renal response and 2571 (IQR 2896) mg/g in patients with no response at one year (*P* = 0.003).

### Renal function at one year was the main predictor of progression to CKD

During follow-up, 38 patients developed CKD: 32 with PLN, three with MLN and three with mixed LN. Another four patients already had CKD when the diagnosis of LN was made (three patients with PLN and one with mixed LN), and these were excluded from the analysis. Table 3 shows that patients that developed CKD were more likely to be older (*P* = 0.011), male (*P* = 0.013), have neurologic involvement (*P* = 0.015), have diabetes (*P* = 0.050) and were less likely to have anti-RNP antibodies (*P* = 0.031). They had significantly higher creatinine levels at diagnosis and a significantly poorer response to treatment (renal response 18% *vs* 77%, *P* < 0.001). Azathioprine was used for induction of remission in 20% of these patients (and only in 5% of the patients that did not progress to CKD). Death was more frequent among patients with CKD.

**Table 3.** Comparison between patients that developed CKD and the ones that did not

	CKD N = 38	No CKD N = 205	*P*
Male sex, N (%)	10 (26)	23 (11)	**0.013**
Time SLE-LN(y), median (IQR)	2.0 (5.0)	1.0 (5.0)	0.533
Initial-onset LN, N (%)	15 (41)	93 (46)	0.570
Age LN diagnosis(y), median (IQR)	38 (24)	30 (16)	**0.011**
Ethnicity			
White European, N (%)	32 (84)	182 (89)	
African ancestry, N (%)	3 (8)	20 (10)	**0.022**
Other, N (%)	3 (8)	2 (1)	
Histology			
Membranous, N (%)	3 (8)	40 (20)	0.092
Proliferative, N (%)	32 (84)	159 (78)	
Mixed, N (%)	3 (8)	6 (3)	
Neurologic involvement, N (%)	7 (19)	11 (6)	**0.015**
Ever positive anti-RNP antibodies, N (%)	5 (15)	64 (34)	**0.031**
Induction treatment with azathioprine, N (%)	5 (20)	8 (5)	**0.014**
Comorbidities			
Diabetes (ever), N (%)	4 (11)	5 (3)	**0.050**
Hypertension (ever), N (%)	13 (34)	41 (23)	0.128
Antiphospholipid syndrome, N (%)	2 (5)	13 (7)	1.000
Creatinine at LN diagnosis, median (IQR)	1.12 (0.42)	0.80 (0.26)	<0.001*
eGFR at LN diagnosis, mean ± SD	65 ± 30	104 ± 29	<0.001*
Creatinine at 1 year, median (IQR)	1.17 (0.58)	0.71 (0.22)	<0.001*
eGFR at 1 year, mean ± SD	65 ± 31	111 ± 23	<0.001*
Renal response 1 year, N (%)	4 (18)	95 (77)	<0.001*
Complete renal response 1 year, N (%)	4 (18)	88 (71)	<0.001*
Death, N (%)	7 (18)	9 (4)	**0.005**

Our analyses included all the variables related to clinical manifestations, laboratory and treatment; however, in order to simplify the table, we have presented only the variables from these three aspects that are significantly different between groups. Creatinine is presented in mg/dl and eGFR in mL/min/1.73 m^2^.

*P* values < 0.05 are presented in bold.

* *P* values <0.001 remain significant after applying the Bonferroni correction.

Cumulative survival free of CKD at 5, 10, 15 and 20 years was 98, 98, 91 and 91% for MLN, and 90, 81, 80 and 75% for PLN, respectively (Fig. S1, available at *Rheumatology* online, graph on Histology). Despite this numerical difference, the difference in the survival curves between MLN and PLN did not reach statistical significance (*P* = 0.091). There was also no difference in the survival curves between class III and class IV, within PLN (*P* = 0.565).

Table 4 presents the significant predictors of CKD found on univariable Cox regression (Table S1, available at *Rheumatology* online shows the full list of variables tested), which are in agreement with the results shown in Table 3. In the ROC analysis (Fig. S2, available at *Rheumatology* online), eGFR at one year after the renal biopsy had an AUC of 0.87 [95% CI 0.77–0.97] *P* < 0.001 as a predictor of CKD, and we identified the cut-off of 75 mL/min as having 77% sensitivity and 94% specificity. eGFR ≤75 at one year showed then the highest hazard ratio (HR) for the association with CKD: HR 22.86 [95% CI 8.38–62.36], *P* < 0.001.

**Table 4.** Hazard ratios (HR) for possible predictors of CKD, identified by univariable Cox regression analysis

Univariable Cox regression	B coefficient	HR [95%CI]	*P*
No renal response 1 year (*N* = 143)	2.33	10.32 [3.49–30.56]	**<0.001**
eGFR at 1 y (continuous variable) (*N* = 160)	-0.06	0.95 [0.93–0.96]	**<0.001**
eGFR ≤75 at 1 y (*N* = 160)	3.13	22.86 [8.38–62.36]	**<0.001**
Creatinine at 1 y (continuous variable) (*N* = 160)	0.40	1.50 [1.26–1.78]	**<0.001**
Creatinine at 1 y ≥ 1.07 (*N* = 160)	3.12	22.60 [8.75–58.37]	**<0.001**
eGFR at LN diagnosis (continuous variable) (*N* = 176)	-0.04	0.96 [0.95–0.97]	**<0.001**
eGFR ≤75 at LN diagnosis (*N* = 176)	2.41	11.09 [4.32–28.48]	**<0.001**
Creatinine at LN diagnosis (continuous variable) (*N* = 176)	0.38	1.46 [1.23–1.72]	**<0.001**
Creatinine at LN diagnosis ≥0.99 (*N* = 176)	2.26	9.58 [3.52–26.10]	**<0.001**
Mixed LN (ref. membranous) (*N* = 243)	2.20	9.04 [1.78–45.97]	**0.008**
Age at LN diagnosis (continuous variable) (*N* = 243)	0.04	1.04 [1.02–1.07]	**0.001**
Age at LN diagnosis ≥36 (*N* = 243)	1.33	3.79 [1.92–7.48]	**<0.001**
Induction with azathioprine (*N* = 197)	1.20	3.34 [1.24–8.96]	**0.017**
Neurologic involvement (*N* = 226)	1.08	2.95 [1.29–6.76]	**0.011**
Anti-RNP (*N* = 221)	-1.00	0.37 [0.14–0.96]	**0.041**
Male sex (*N* = 243)	0.69	2.00 [0.96–4.17]	0.065
Hypertension (*N* = 220)	0.64	1.90 [0.96–3.74]	0.064

This table presents only the variables with a *P*-value of <0.1. The full list of variables tested and respective HR is included in the supplementary materials (Table S1, available at *Rheumatology* online).

*P* values < 0.05 are presented in bold.

When adjusted for eGFR at one year (Table 5), induction treatment with azathioprine and mixed LN remained significantly associated with progression to CKD; however, for mixed LN, the confidence interval is considerably larger due to the very small number of cases included; therefore, this result should be interpreted with caution (Table S2, available at *Rheumatology* online shows the hazard ratios for eGFR at 1 year, adjusted for each of the variables in Table 5).

**Table 5.** Hazard ratios for possible predictors of CKD, adjusted for eGFR at 1 year (continuous variable) and eGFR ≤75 at 1 year (multivariable Cox regression)

Adjusted for eGFR at 1y (continuous variable)	B coefficient	HR [95%CI]	*P*
Histologic class—Mixed LN (ref MLN)	0.96	2.62 [0.21–33.15]	0.457
Age LN diagnosis (continuous variable)	0.03	1.03 [1.00–1.06]	0.060
Age LN diagnosis ≥36	0.88	2.42 [0.89–6.59]	0.085
Induction AZA	**1.53**	**4.61 [1.49–14.29]**	**0.008**
Neurologic involvement	1.08	2.95 [0.96–9.08]	0.060
Anti-RNP	–0.61	0.54 [0.15–1.91]	0.342
Male sex	0.21	1.24 [0.43–3.57]	0.696
Hypertension	0.12	1.13 [0.40–3.14]	0.820

**Adjusted for eGFR ≤75 at 1 y**	**B coefficient**	**HR [95%CI]**	***P***

Histologic class—Mixed LN (ref MLN)	**2.64**	**13.99 [1.36–143.86]**	**0.026**
Age at LN diagnosis (continuous variable)	0.023	1.02 [0.99–1.06]	0.177
Age at LN diagnosis ≥36	0.497	1.65 [0.54–5.03]	0.383
Induction with AZA	1.05	2.86 [0.93–8.83]	0.067
Neurologic involvement	0.98	2.66 [0.86–8.22]	0.089
Anti-RNP	–0.71	0.49 [0.14–1.71]	0.266
Male sex	0.91	2.47 [0.95–6.42]	0.063
Hypertension	0.52	1.69 [0.70–4.06]	0.242

Note: *N* = 160. Due to the small number of patients developing CKD, multivariable models could not include more than two variables at a time. Because eGFR at one year showed such a strong result in the univariable analysis, we decided that this variable should be included in the multivariable model. Therefore, we tested each variable together with eGFR at one year. As the variable ‘renal response’ includes the eGFR, these variables are colinear and therefore cannot be included in the same model. The hazard ratios for eGFR at 1 year, adjusted for each of the variables in Table 5, can be found in the supplementary materials (Table S2, available at *Rheumatology* online).

*P* values < 0.05 are presented in bold.

### Ten-year renal survival was 96% and patient survival was 93%

Ten patients (4%) progressed to ESRD during follow-up: seven with PLN (five with class IV, one with class III and one with no information on class), two with mixed LN and one patient with MLN. Interestingly, this patient had been diagnosed with membranous nephropathy one year before the diagnosis of SLE. Overall cumulative renal survival at 5, 10, 15 and 20 years was 97, 96, 94 and 94%, respectively (Fig. S3, available at *Rheumatology* online).

Sixteen out of 260 patients (6%) died during follow-up: 15 patients with PLN and one patient with MLN. Causes of death were infection (*N* = 6), cancer (*N* = 3), cardiovascular (*N* = 1) and alveolar haemorrhage (*N* = 1). In five patients, the cause of death was uncertain or unknown. Cumulative patient survival figures at 5, 10, 15 and 20 years were 97, 93, 91 and 88%, respectively (Fig. S4, available at *Rheumatology* online).

## Discussion

We have studied a multicentre Portuguese cohort of LN patients with a long follow-up. To our knowledge, this is the first multicentre study directly comparing patients with PLN *vs* MLN. Our results show that patients with PLN have significantly higher creatinine levels at the time of the renal biopsy, more frequent positivity for anti-dsDNA antibodies and lower complement levels than those with MLN. At one year, 62% and 5% of the patients achieved complete and partial renal response, respectively, according to the EULAR/ERA-EDTA definition. A total of 33% of the patients had no response by this timepoint. Having an eGFR ≤75 ml/min/1.73 m^2^ at one year was the single best predictor of progression to CKD.

Our previous study with 187 patients with biopsy-proven LN from a single centre in the UK had shown very similar results regarding the differences between PLN and MLN. In that study, median anti-dsDNA levels at the time of biopsy were six times higher in the PLN group compared with the MLN group [21]. In the current study, we analysed anti-dsDNA antibodies as a categorical variable (positive/negative) because the quantification assay was not the same in every centre, so levels of antibodies were not comparable between different centres. We showed a highly significant difference in the prevalence of anti-dsDNA positivity at the time of diagnosis of LN: 48% in MLN *vs* 92% in PLN (*P* < 0.001).

A few recent studies have compared patients in whom LN was present at the onset of SLE (initial-onset LN) with patients developing LN later during the course of their SLE [29–32]. We did not find any difference between these patients regarding progression to CKD.

Renal response rates in our study are similar to the ones reported in a recent paper by Moroni and collaborators [33]. These authors studied 381 patients with biopsy-proven LN and showed that, 12 months after the beginning of treatment, 58, 26 and 16% of the patients achieved complete, partial and no response, respectively. Lack of response at 12 months predicted CKD with a HR 5.60 [95%CI 3.23–9.73], *P* < 0.001. Our results are comparable: lack of renal response at one year had a HR 10.32 [3.49–30.56], *P* < 0.001, as a predictor of CKD.

Some important studies, notably the ones analysing long term outcomes of the Euro-Lupus and the MAINTAIN Nephritis cohorts, found proteinuria at one year to be the most important prognostic marker [12, 34]. In our previous study, uPCR above 42 mg/mmol (∼420 mg/g) and eGFR below 76 ml/min/1.73 m^2^, one year after the diagnosis of LN, were the strongest predictors of progression to ESRD [21]. However, in our current study, although the level of proteinuria at time of diagnosis predicted EULAR/ERA-EDTA response at one year, neither proteinuria at diagnosis nor at one year were associated with long-term progression to CKD. We hypothesise that this lack of association between proteinuria at one year and progression CKD may be due to our small sample size; however, we should also keep in mind that clinical markers of response, such as proteinuria, have some limitations. In fact, several studies using per-protocol kidney biopsies, performed after the initial phase of immunosuppression, have showed substantial disagreement between clinical and histopathological evaluation [35–39].

An eGFR of 75 mL/min/1.73 m^2^ is likely to be a highly significant result in a patient with an expected GFR closer to being over 100 mL/min/1.73 m^2^. A level of 75 will represent significant glomerular loss has already occurred. Our patients with an eGFR ≤75 ml/min/1.73 m^2^ at one year, with progression to CKD, probably correspond to those with ongoing rapid loss of eGFR found in the recent study by Weeding *et al.* [39] We believe it is of utmost importance to prevent further LN flares in those patients, as one renal flare would mean a substantial amount of further nephron loss [40].

This was a multicentre nationwide study in real-world patients with biopsy-proven LN and a long follow-up. The Rheumatic Disease Portuguese Registry – Reuma.pt started in 2008 and the patients with SLE began to be included prospectively in the registry in 2012; therefore, for patients diagnosed with LN before 2012 (*N* = 160, including 26 patients with MLN, 130 patients with PLN and four patients with mixed LN), there is a combination of retrospective and prospectively collected data, and this is a limitation of our study.

We have some missing data, mainly related to laboratory data both at the time of biopsy and at one year. These patients could not be included in the Cox regression analyses investigating the effect of those specific laboratory parameters. Most patients with missing data were diagnosed before the existence of the registry and had a longer time of follow-up; however, the characteristics of the patients with missing data do not differ significantly from the ones included in the analysis (Table S3, available at *Rheumatology* online), therefore, we believe this missing data did not significantly bias our study. Furthermore, our results were still consistent with the ones from other recent studies.

Another limitation of our study is the lack of data regarding the activity and chronicity indices in the kidney biopsies, as these may influence the renal outcomes. Furthermore, biopsy reports were not reviewed by a central pathologist.

In conclusion, serological profiles are significantly different between patients with MLN and PLN, which might suggest a different pathophysiology. Future studies on pathogenesis of LN should probably look at these two groups separately. Proteinuria levels did not predict LN histologic class in our cohort.

Finally, renal function at year one after LN diagnosis appeared to be the best predictor of progression to CKD. Patients with an eGFR ≤75 ml/min/1.73 m^2^ at one year, in particular, should receive special attention. A repeated renal biopsy may be useful to help distinguish between ongoing active disease (needing more aggressive immunosuppression) from irreversible damage. All patients should be closely monitored to ensure that any flares are rapidly detected and treated, in order to avoid further loss of GFR. Furthermore, tight blood pressure control, treatment with ACEi and, in those with CKD and mild proteinuria, the use of sodium–glucose cotransporter-2 inhibitors [41], may be extremely important to improve outcomes.

## Supplementary material

Supplementary material is available at *Rheumatology* online.

## Supplementary Material

keae236_Supplementary_Data

## Data Availability

The data underlying this article will be shared upon reasonable request to the corresponding author.
