# Supplementary material for: Risk of chronic kidney disease in 260 patients with lupus nephritis: analysis of a nationwide multicentre cohort with up to 35 years of follow-up
Source: Rheumatology (Oxford). 2024 Apr 22;64(3):1201–9. doi: 10.1093/rheumatology/keae236 (PMC11879298; doi:10.1093/rheumatology/keae236)
Supplement: keae236_Supplementary_Data [file keae236_supplementary_data.docx]

**Risk of CKD in 260 patients with membranous and proliferative lupus nephritis - analysis of a nationwide multicentre cohort with up to 35 years of follow-up**

Supplementary data

Table S1: Hazard ratios for possible predictors of CKD, identified by univariable Cox regression analysis.

| **Univariable Cox Regression** | **B coefficient** | **HR [95%CI]** | **p** |
| --- | --- | --- | --- |
| No renal response 1 year (N=143) | 2.33 | 10.32 [3.49-30.56] | **<0.001** |
| eGFR at 1y (continuous variable) (N=160) | -0.06 | 0.95 [0.93-0.96] | **<0.001** |
| eGFR ≤ 75 at 1y (N=160) | 3.13 | 22.86 [8.38-62.36] | **<0.001** |
| Creatinine 1y (continuous variable) (N=160) | 0.40 | 1.50 [1.26-1.78] | **<0.001** |
| Creatinine 1y ≥ 1.07 (N=160) | 3.12 | 22.60 [8.75-58.37] | **<0.001** |
| eGFR at LN diagnosis (continuous variable) (N=176) | -0.04 | 0.96 [0.95-0.97] | **<0.001** |
| eGFR ≤ 75 at diagnosis (N=176) | 2.41 | 11.09 [4.32-28.48] | **<0.001** |
| Creatinine diagnosis (continuous variable) (N=176) | 0.38 | 1.46 [1.23-1.72] | **<0.001** |
| Creatinine diagnosis ≥ 0.99 (N=176) | 2.26 | 9.58 [3.52-26.10] | **<0.001** |
| Mixed LN (ref. membranous) (N=243) | 2.20 | 9.04 [1.78-45.97] | **0.008** |
| Age LN diagnosis (continuous variable) (N=243) | 0.04 | 1.04 [1.02-1.07] | **0.001** |
| Age LN diagnosis ≥ 36 (N=243) | 1.33 | 3.79 [1.92-7.48] | **<0.001** |
| Induction azathioprine (N=197) | 1.20 | 3.34 [1.24-8.96] | **0.017** |
| Neurologic involvement (N=226) | 1.08 | 2.95 [1.29-6.76] | **0.011** |
| Anti-RNP (N=221) | -1.00 | 0.37 [0.14-0.96] | **0.041** |
| Male sex (N=243) | 0.69 | 2.00 [0.96-4.17] | 0.065 |
| Hypertension (N=220) | 0.64 | 1.90 [0.96-3.74] | 0.064 |
| African ancestry (N=241) | 0.32 | 1.38 [0.42-4.58] | 0.597 |
| Date of LN diagnosis (N=243) | 0.00 | 1.00 [0.96-1.05] | 0.859 |
| Diabetes (N=220) | 0.83 | 2.29 [0.80-6.53] | 0.121 |
| Antiphospholipid syndrome (N=220) | -0.36 | 0.70 [0.17-2.90] | 0.619 |
| Antiphospholipid antibodies (N=187) | 0.24 | 1.27 [0.58-2.77] | 0.557 |
| Anti-Ro (N=224) | -0.30 | 0.75 [0.35-1.57] | 0.437 |
| Anti-Sm (N=227) | -0.48 | 0.62 [0.24-1.62] | 0.331 |
| Positive anti-dsDNA at diagnosis (N=162) | 0.46 | 1.58 [0.37-6.78] | 0.540 |
| C3 level at LN diagnosis (N=173) | 0.59 | 1.80 [0.44-4.33] | 0.415 |
| C4 level at LN diagnosis (N=161) | 4.22 | 67.79 [0.91-5072] | 0.055 |
| SLEDAI at diagnosis (N=101) | 0.07 | 1.08 [0.98-1.18] | 0.114 |
| uPCR at LN diagnosis (N=166) | 0.00 | 1.00 [1.00-1.00] | 0.551 |
| uPCR at 1y (N=146) | 0.00 | 1.00 [1.00-1.00] | 0.556 |
| Haematological involvement (N=234) | 0.66 | 1.94 [0.80-4.70] | 0.143 |
| Induction MMF (N=197) | -0.10 | 0.91 [0.39-2.13] | 0.824 |
| Induction cyclophosphamide (N=197) | -0.42 | 0.66 [0.27-1.62] | 0.361 |
| Induction calcineurin inhibitor (N=197) | -0.49 | 0.61 [0.08-4.54] | 0.631 |
| Induction rituximab (N=197) | 1.03 | 2.81 [0.37-21.24] | 0.316 |
| Use of antimalarials (N=237) | 0.62 | 1.87 [0.66-5.29] | 0.242 |
| Use of steroids (N=229) | 0.65 | 1.92 [0.73-5.00] | 0.184 |
| Use of ACEi / ARB (N=234) | 0.31 | 1.36 [0.62-2.99] | 0.447 |
| Use of NSAIDs (N=235) | -0.20 | 0.82 [0.25-2.67] | 0.740 |
| More than 1 renal biopsy (N=243) | 0.01 | 1.01 [0.44-2.32] | 0.973 |
| Proliferative LN (ref. membranous) (N=243) | 0.98 | 2.67 [0.81-8.80] | 0.107 |

Table S2: Hazard ratios for eGFR at 1y (continuous variable) and eGFR ≤ 75 at 1y, adjusted for each of the variables in the rows

| HR for eGFR at 1y (continuous variable) | **B coefficient** | **HR [95%CI]** | **p** |
| --- | --- | --- | --- |
| Histologic class - Mixed LN (ref memb) | -0.06 | 0.95 [0.93-0.96] | **<0.001** |
| Age LN diagnosis (continuous variable) | -0.06 | 0.95 [0.93-0.96] | **<0.001** |
| Age LN diagnosis ≥ 36 | -0.06 | 0.95 [0.93-0.96] | **<0.001** |
| Induction AZA | -0.08 | 0.93 [0.90-0.95] | **<0.001** |
| Neurologic involvement | -0.06 | 0.95 [0.93-0.96] | **<0.001** |
| Anti-RNP | -0.06 | 0.95 [0.93-0.97] | **<0.001** |
| Male sex | -0.06 | 0.95 [0.93-0.96] | **<0.001** |
| Hypertension | -0.06 | 0.95 [0.93-0.97] | **<0.001** |
| HR for eGFR ≤ 75 at 1y | **B coefficient** | **HR [95%CI]** | **p** |
| Histologic class - Mixed LN (ref memb) | 3.13 | 22.96 [8.32-63.38] | **<0.001** |
| Age LN diagnosis (continuous variable) | 2.82 | 16.70 [5.53-50.46] | **<0.001** |
| Age LN diagnosis ≥ 36 | 2.90 | 18.17 [5.96-55.41] | **<0.001** |
| Induction AZA | 3.39 | 29.70 [8.51-103.61] | **<0.001** |
| Neurologic involvement | 3.07 | 21.55 [7.79-59.61] | **<0.001** |
| Anti-RNP | 2.97 | 19.51 [6.98-54.49] | **<0.001** |
| Male sex | 3.11 | 22.45 [8.22-61.34] | **<0.001** |
| Hypertension | 2.99 | 19.94 [7.26-54.78] | **<0.001** |

Note: N=160. Due to the small number of patients developing CKD, multivariable models could not include more than two variables at a time. Because eGFR at one year showed such a strong result in the univariable analysis, we decided that this variable should be included in the multivariable model. Therefore, we tested each variable together with eGFR at one year. As the variable “renal response” includes the eGFR, these variables are colinear and therefore cannot be included in the same model.

Table S3: Comparison between patients with and without missing data regarding laboratory parameters at the time of the renal biopsy.

|  | | **Included cases** | **Missing cases** | **p** |
| --- | --- | --- | --- | --- |
| **Total**, N | | 169 | 91 |  |
| **Females,** N (%) | | 144 (85) | 81 (89) | 0.391 |
| **Ethnicity** | **White European**, N (%) | 149 (89) | 75 (85) | 0.445 |
|  | **African ancestry**, N (%) | 17 (10) | 10 (11) |  |
|  | **Other**, N (%) | 2 (1) | 3 (3) |  |
| **Time SLE-LN**(y), median (IQR) | | 1 (5) | 1 (5) | 0.899 |
| **Initial-onset LN**, N (%) | | 75 (44) | 37 (42) | 0.666 |
| **Age LN diagnosis**(y), median (IQR) | | 31 (19) | 30 (15) | 0.364 |
| **SLEDAI-2K at LN diagnosis,** median (IQR) | | 16 (10) | 12 (8) | 0.121 |
| **Histology** | **Proliferative**, N (%) | 135 (80) | 68 (75) | 0.209 |
|  | **Membranous**, N (%) | 26 (15) | 21 (23) |  |
|  | **Mixed**, N (%) | 8 (5) | 2 (2) |  |
| **Other manifestations**, N (%) | |  |  |  |
| Mucocutaneous | | 115 (68) | 63 (69) | 0.845 |
| Joint involvement | | 118 (71) | 61 (68) | 0.632 |
| Serositis | | 54 (33) | 33 (38) | 0.447 |
| Neurologic | | **7 (4)** | **11 (13)** | **0.013** |
| Haematological | | 128 (78) | 61 (73) | 0.342 |
| **Historical serology (ever positive), N (%)** | |  |  |  |
| ANA | | 162 (96) | 89 (99) | 0.427 |
| Low Complement | | 148 (90) | 71 (88) | 0.536 |
| Anti-dsDNA | | 157 (94) | 78 (88) | 0.113 |
| Anti-Sm | | 37 (23) | 22 (27) | 0.525 |
| Anti-Ro | | 56 (35) | 28 (36) | 0.918 |
| Anti-La | | 24 (15) | 10 (13) | 0.639 |
| Anti-RNP | | 49 (31) | 25 (33) | 0.846 |
| Antiphospholipid antibodies | | 41 (30) | 28 (43) | 0.077 |
| **Antiphospholipid Syndrome**, N (%) | | 8 (5) | 8 (10) | 0.143 |
| **Hypertension (ever)**, N (%) | | 40 (26) | 19 (25) | 0.831 |
| **Diabetes (ever)**, N (%) | | 4 (3) | 5 (7) | 0.164 |
| **Induction treatment**, N (%) | |  |  |  |
| Mycophenolate | | 79 (52) | 23 (40) | 0.134 |
| Cyclophosphamide | | 46 (30) | 20 (35) | 0.504 |
| Azathioprine | | 9 (6) | 5 (9) | 0.535 |
| Rituximab | | 5 (3) | 1 (2) | 1.000 |
| Calcineurin inhibitors | | 9 (6) | 4 (7) | 0.754 |
| **Maintenance treatment**, N (%) | |  |  |  |
| Mycophenolate | | **95 (63)** | **26 (47)** | **0.044** |
| Cyclophosphamide | | **6 (4)** | **9 (16)** | **0.005** |
| Azathioprine | | 24 (16) | 7 (13) | 0.574 |
| Rituximab | | 2 (1) | 0 (0) | 1.000 |
| Calcineurin inhibitors | | 10 (7) | 5 (9) | 0.551 |
| **Use of antimalarials**, N (%) | | 146 (96) | 52 (90) | 0.096 |
| **Use of steroids**, N (%) | | 137 (92) | 49 (91) | 0.778 |
| **Use of ACEi / ARB**, N (%) | | 116 (77) | 41 (73) | 0.590 |
| **Use of NSAIDs (ever)**, N (%) | | 14 (9) | 8 (14) | 0.291 |
| **SLEDAI-2K at one year,** median (IQR) | | 4 (6) | 4 (3) | 0.720 |
| **Time follow-up since LN**, median (IQR) | | **7 (8)** | **12 (16)** | **<0.001** |
| **More than 1 renal biopsy,** N (%) | | 27 (16) | 15 (17) | 0.916 |
| **Different class in subs. biopsy,** N (%) | | 6 (22) | 6 (40) | 0.292 |
| **CKD after LN diagnosis**, N (%) | | **20 (12)** | **18 (23)** | **0.024** |
| **ESRD after LN diagnosis – total, N** (%) | | 4 (2) | 6 (7) | 0.082 |
| **ESRD – subgroup transplant, N** (%) | | 2 (50) | 4 (67) | 1.000 |
| **Deaths, N** (%) | | 11 (7) | 5 (6) | 0.745 |

ACEi/ARB: angiotensin-converting enzyme inhibitors/ angiotensin receptor blockers; NSAIDs: nonsteroidal anti-inflammatory drugs; CKD: Chronic kidney disease (eGFR<60 mL/min/1.73m^2^) y: years; ESRD: end-stage renal disease; LN: lupus nephritis; SLEDAI: Systemic Lupus Erythematosus Disease Activity Index


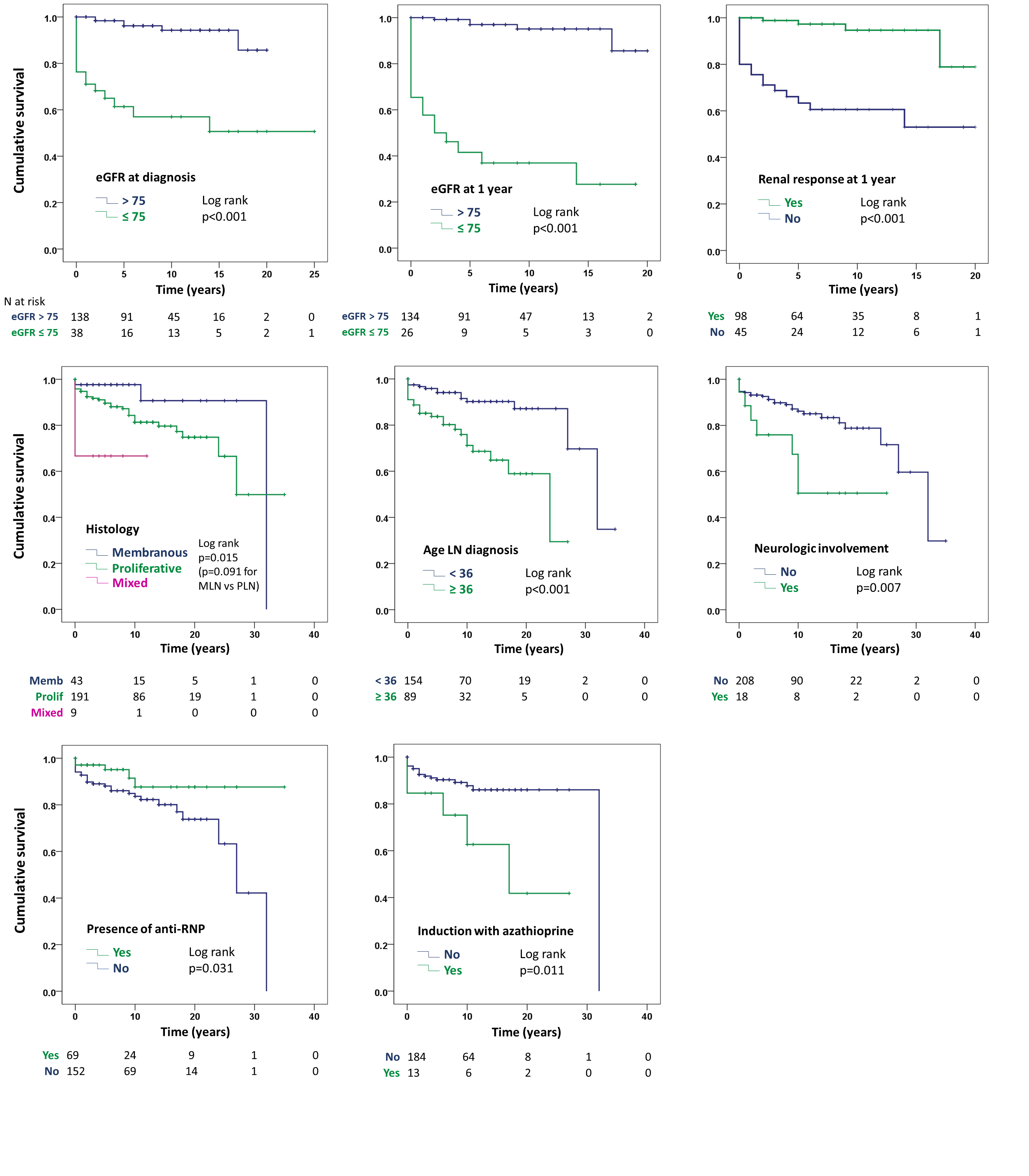


Figure S1: Kaplan-Meier curves showing cumulative survival free of CKD for different groups of patients with LN


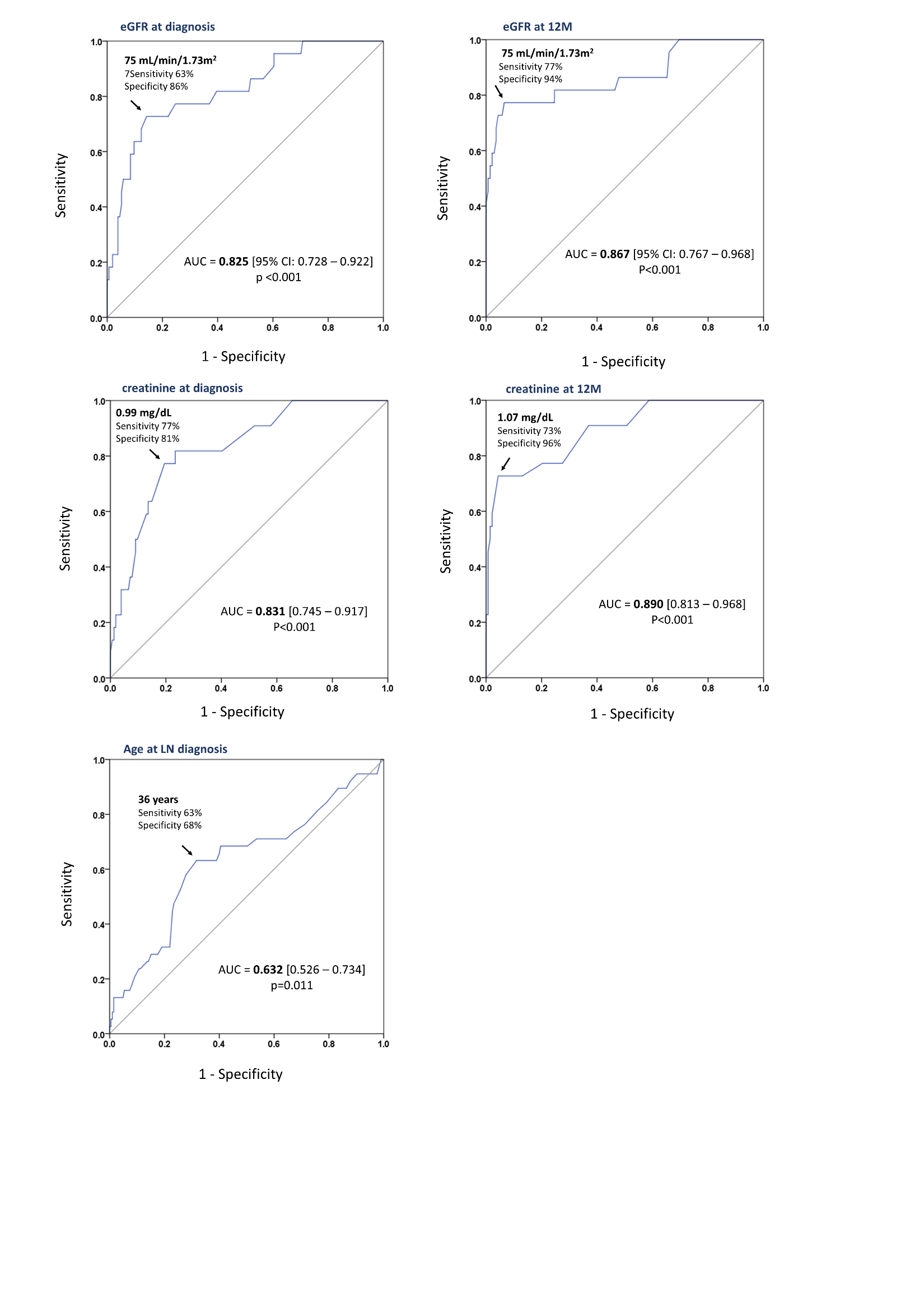


Figure S2: ROC curves for significant predictors of CKD on COX regression analysis.


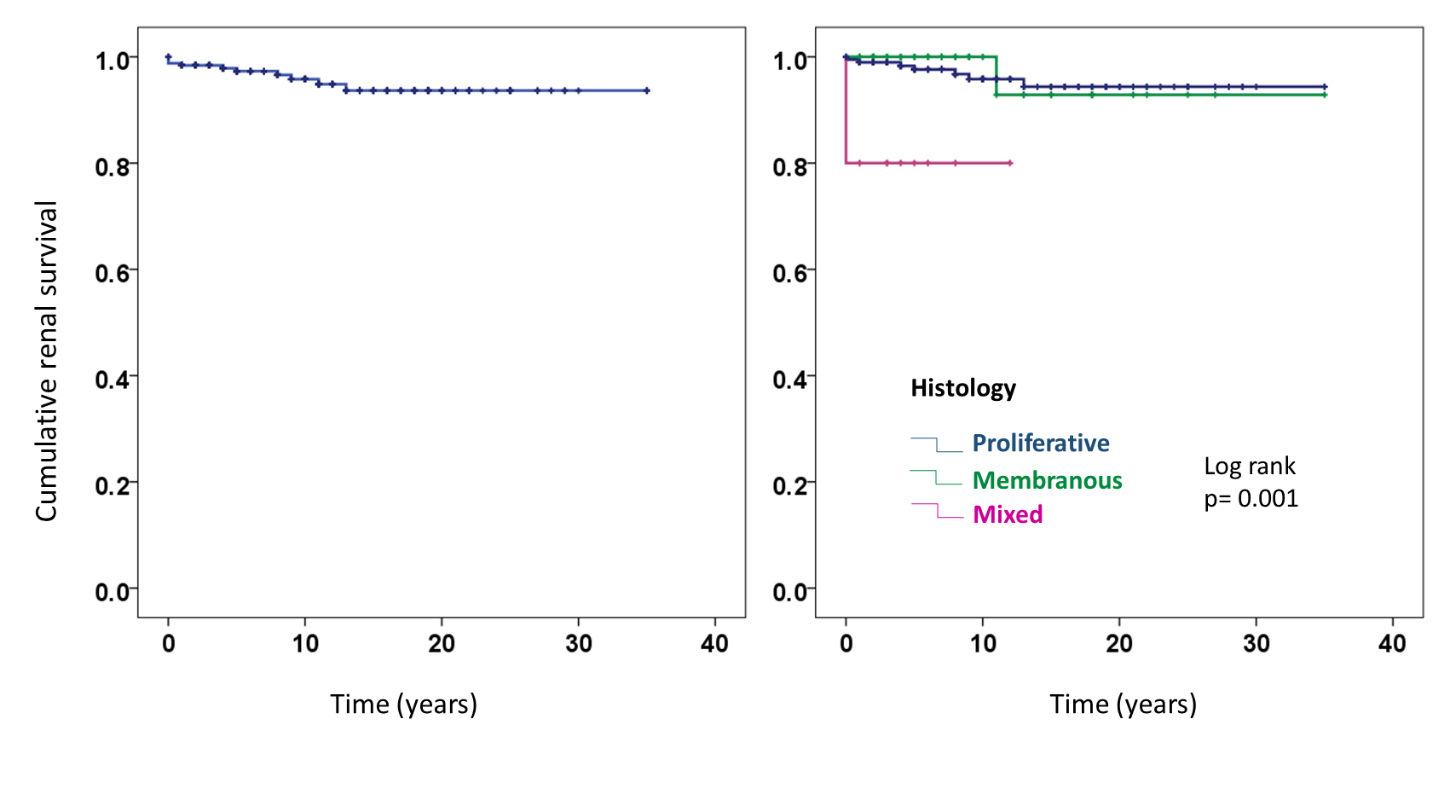


Figure S3: Overall cumulative renal survival on the left and cumulative renal survival according to histology on the first renal biopsy on the right.


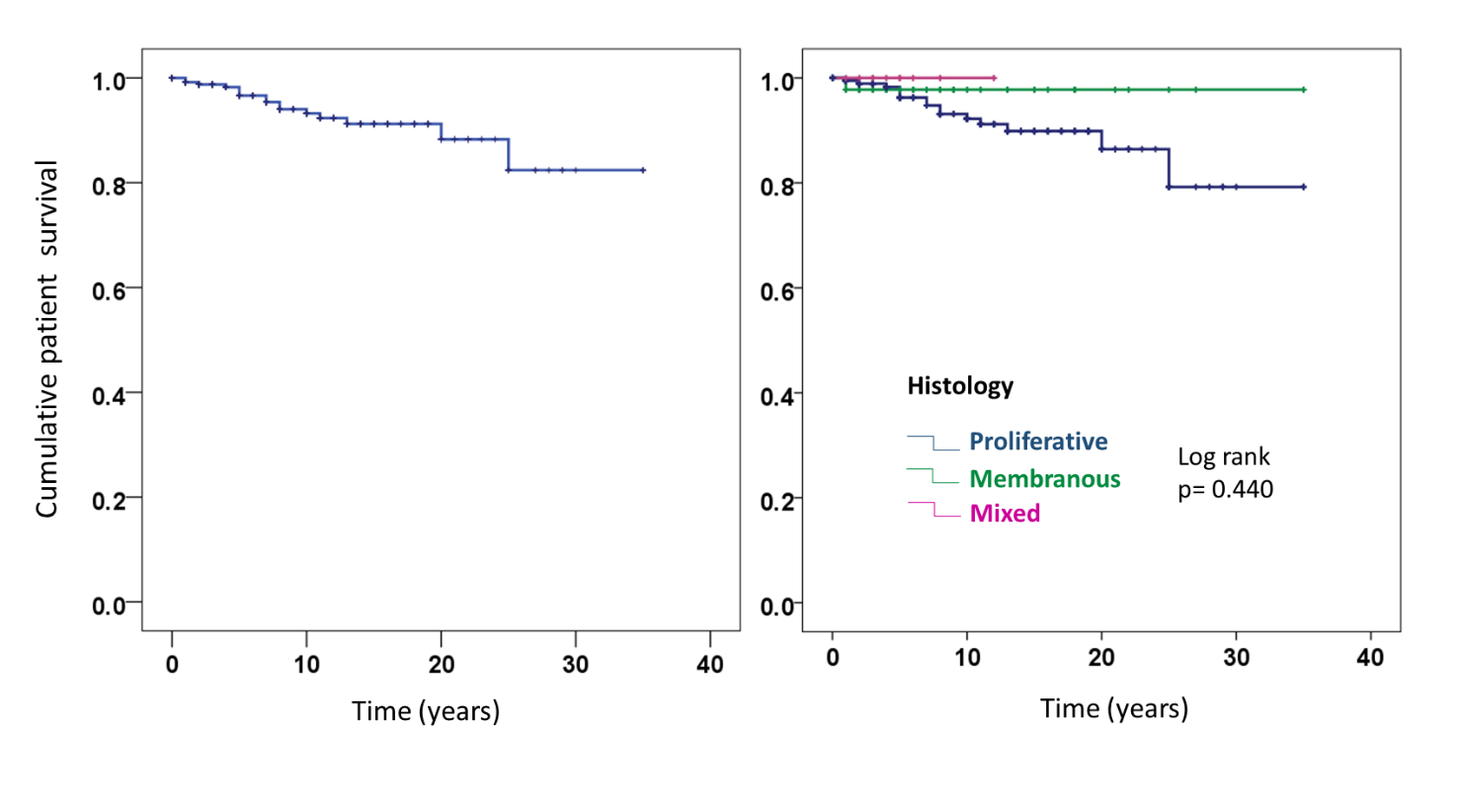


Figure S4: Overall cumulative patient survival on the left and cumulative patient survival according to histology on the first renal biopsy on the right.
